# Medicare and Medicaid Behavioral Health Service Use Among Dual-Eligible Special Needs Plan Enrollees

**DOI:** 10.1001/jamanetworkopen.2025.54246

**Published:** 2026-01-15

**Authors:** Hyunjee Kim, Sara Edelstein, Angela Senders, Maanyatha Cheekati, Stephan R. Lindner, K. John McConnell, Jeah Jung

**Affiliations:** 1 Center for Health Systems Effectiveness, Oregon Health and Science University, Portland; 2 Department of Health Administration and Policy, College of Public Health, George Mason University, Fairfax, Virginia

## Abstract

This cross-sectional study investigates the prevalence of behavioral health services among Medicare and Medicaid enrollees in dual-eligible special needs plans and the distribution paid for by each program.

## Introduction

Individuals with both Medicare and Medicaid coverage (dual-eligible enrollees) are eligible to receive behavioral health (BH) services through both programs.^1^ However, navigating 2 separate systems can be challenging because enrollees may not know which program covers which BH services or how coverage changes by care setting or clinician. To address these challenges, the CMS has increasingly emphasized integrating Medicare and Medicaid BH services for dual-eligible enrollees using dual-eligible special needs plans (D-SNPs).^2^

Integration efforts via D-SNPs presume that both Medicare and Medicaid play significant roles in delivering BH services. However, the breakdown of BH service use across the programs remains largely unknown. Focusing on D-SNP enrollees, we describe the prevalence of BH conditions and the distribution of BH services paid by Medicare vs Medicaid.

## Methods

This cross-sectional study followed the STROBE reporting guideline and was approved by the Oregon Health and Science University Institutional Review Board with consent waived because seeking informed consent from all patients was not feasible and the risk to participants was minimal. We analyzed 2021 Medicare Advantage encounter records and Medicaid claims of D-SNP enrollees. First, we calculated the prevalence of 3 types of BH conditions (mild to moderate mental illness, serious mental illness, and substance use disorder) separately for enrollees younger than 65 years and aged 65 years and older. Second, among enrollees with BH conditions, we described BH service use (services with a BH condition as the primary diagnosis) paid by Medicare vs Medicaid in emergency department, inpatient, residential treatment facility, in-person outpatient, and telehealth settings (eMethods in Supplement 1). Data were analyzed from April through October 2025 using R version 4.5.1 (R Project for Statistical Computing).

## Results

Among 652 250 enrollees younger than 65 years (58.7% female; mean [SD] age, 50.9 [10.7] years), 401 186 (61.5%) had 1 or more BH condition, including 250 631 with mild to moderate mental illness (38.4%), 127 624 with serious mental illness (19.6%), and 114 247 with substance use disorder (17.5%) (Table). Among 955 022 enrollees aged 65 years or older (65.2% female; mean [SD] age, 73.7 [7.4] years), 407 909 (42.7%) had 1 or more BH condition.

**Table.** Prevalence of Behavioral Health Conditions

Age group, y	Enrollees, No. (%)
**0-64 (n = 652 250)**	
No behavioral health condition	251 064 (38.5)
Mild to moderate mental illness	250 631 (38.4)
Without substance use disorder	199 125 (30.5)
With substance use disorder	51 506 (7.9)
Serious mental illness	127 624 (19.6)
Without substance use disorder	87 814 (13.5)
With substance use disorder	39 810 (6.1)
Substance use disorder	114 247 (17.5)
Without mild, moderate, or serious mental illness	22 931 (3.5)
With mild, moderate, or serious mental illness	91 316 (14.0)
**≥65 (n = 955 022)**	
No behavioral health condition	547 113 (57.3)
Mild to moderate mental illness	304 579 (31.9)
Without substance use disorder	257 545 (27.0)
With substance use disorder	47 034 (4.9)
Serious mental illness	69 605 (7.3)
Without substance use disorder	53 559 (5.6)
With substance use disorder	16 046 (1.7)
Substance use disorder	96 805 (10.1)
Without mild, moderate, or serious mental illness	33 725 (3.5)
With mild, moderate, or serious mental illness	63 080 (6.6)

Both Medicare and Medicaid paid for BH services in all settings (Figure). Medicare paid most emergency department visits, whereas Medicaid paid most residential treatment days. For inpatient days, in-person outpatient visits, and telehealth visits, both programs paid substantial shares, with Medicare covering a larger share for inpatient days than for in-person outpatient visits and telehealth visits.

**Figure.**
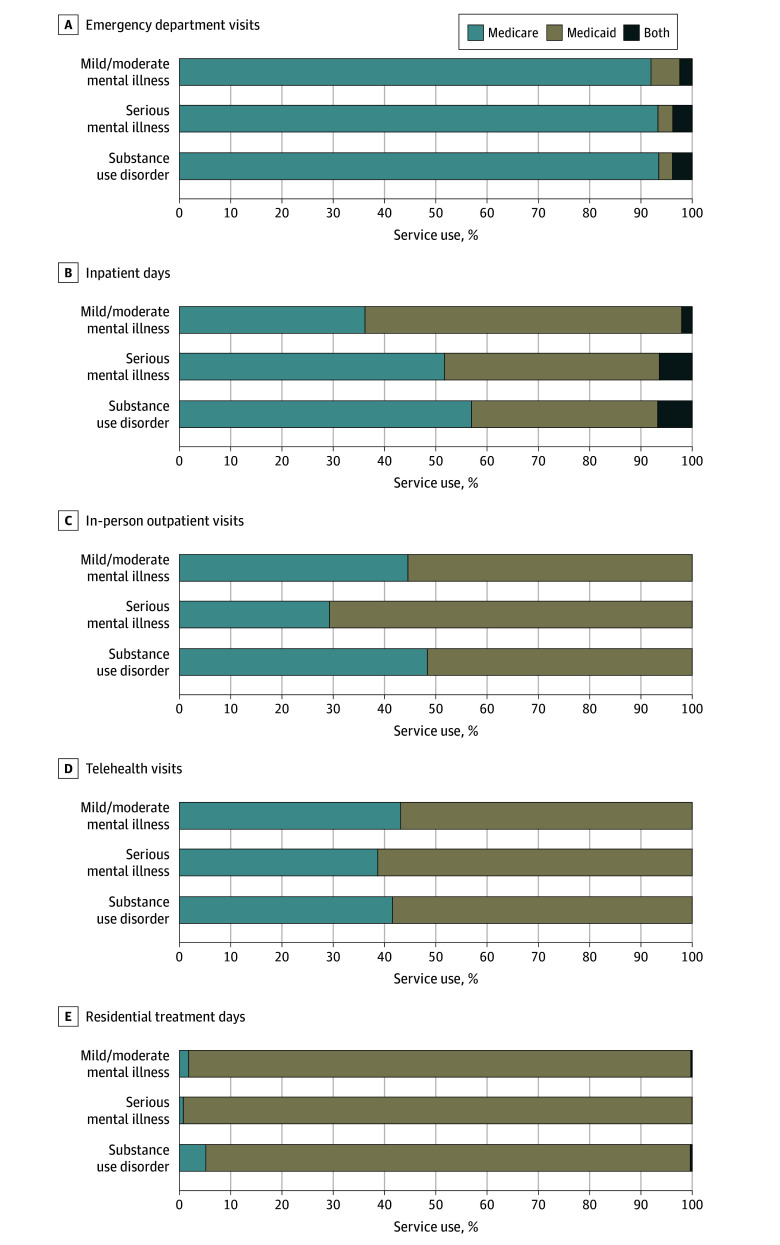
Medicare and Medicaid Payment for Behavioral Health Services Among Dual-Eligible Individuals *Both* indicates visits or days paid by both Medicaid and Medicare for the same service visit or date, where Medicare and Medicaid claims had different diagnosis codes or procedure or revenue codes. See the eMethods in Supplement 1 for further details on determining behavorial health service use, dates of service and payment, and primary payers. This figure included 493 278 beneficiaries, fewer than the 809 095 enrollees with 1 or more behavioral health condition (Table). This is because services reported here require a behavioral health condition listed as the primary diagnosis (or secondary diagnosis for self-harm-related diagnoses) and specific procedure or revenue codes, whereas conditions in the Table have less restrictive definitions, usually requiring only a diagnosis in any position (eMethods in Supplement 1). Overall, 305 730 of 493 278 enrollees (62.0%) used only Medicare-paid services, 36 713 (7.4%) used only Medicaid-paid services, and 150 835 (30.6%) used both programs.

## Discussion

This cross-sectional study found that in 2021, BH conditions were common among D-SNP enrollees younger than 65 years (61.5%) and aged 65 years or older (42.7%). Both Medicare and Medicaid paid BH services across all settings, with their relative share varying by setting.

One prior report^1^ noted that for dual-eligible enrollees, Medicare generally pays for acute care and physician services, while Medicaid fills Medicare’s coverage gaps and provides community-based care. This aligns with our finding that Medicaid paid a substantial share for in-person outpatient visits, which often include community-based BH services, such as case management and recovery supports.

Medicare was the primary payer of acute care, as seen in emergency department visits. However, Medicaid also paid a notable share of inpatient stays. One possible explanation is traditional Medicare’s 190-day lifetime limit for psychiatric hospital care, a restriction most Medicare Advantage plans follow.^3^ Once Medicare benefits are exhausted, Medicaid pays for psychiatric hospital care. Medicare rarely paid for residential treatment, reflecting Medicare’s general exclusion of this service^4^ and leaving Medicaid as the dominant payer.

One limitation of this study is that our analyses used 2021 data. Medicare’s share of outpatient payments may have increased since the 2024 coverage expansion that added intensive outpatient program services^5^ and allows licensed mental health counselors and marriage and family therapists to bill.^6^

Dual-eligible enrollees received BH services paid by both Medicare and Medicaid in all settings, but the relative share varied by setting. These patterns suggest that both Medicare and Medicaid play substantial roles in financing BH care, underscoring the importance of better integrating Medicare and Medicaid BH services.

## Notes

Supplement 1.
eMethods.

eReferences.

Supplement 2.
Data Sharing Statement

## References

[1] ATI Advisory. Improving behavioral healthcare for dual eligible individuals: opportunities and challenges. 2024. Accessed December 3, 2025. https://atiadvisory.com/resources/wp-content/uploads/2024/11/Improving-Behavioral-Health-for-Dual-Eligible-Individuals.pdf

[2] Peña MT, Mohamed M, Biniek JF, Burns A, Cubanski J, Neuman T. The landscape of Medicare and Medicaid coverage arrangements for dual-eligible individuals across states. KFF. October 24, 2024. Accessed January 3, 2025. https://www.kff.org/medicare/the-landscape-of-medicare-and-medicaid-coverage-arrangements-for-dual-eligible-individuals-across-states/

[3] US Government Accountability Office. Behavioral health: information on cost-sharing in Medicare and Medicare Advantage. 2024. Accessed December 3, 2025. https://www.gao.gov/assets/gao-24-106794.pdf

[4] Hurley B, Samuels PN. Congress: close Medicare’s dangerous gaps in coverage for addiction treatment. STAT. May 13, 2024. Accessed August 12, 2025. https://www.statnews.com/2024/05/13/medicare-dangerous-gaps-addiction-treatment-coverage/

[5] Phan M, Triano S, Kelly L, Steinberg D. New changes to behavioral health intensive outpatient program coverage in Medicare. Center for Health Care Strategies. July 2024. Accessed August 12, 2025. https://www.chcs.org/resource/new-changes-to-intensive-outpatient-program-coverage/

[6] Centers for Medicare & Medicaid Services. Marriage and family therapists & mental health counselors. 2024. Accessed August 12, 2025. https://www.cms.gov/medicare/payment/fee-schedules/physician-fee-schedule/marriage-and-family-therapists-mental-health-counselors

