# Supplementary material for: Medicare and Medicaid Behavioral Health Service Use Among Dual-Eligible Special Needs Plan Enrollees
Source: JAMA Netw Open. 2026 Jan 15;9(1):e2554246. doi: 10.1001/jamanetworkopen.2025.54246 (PMC12809357; doi:10.1001/jamanetworkopen.2025.54246)
Supplement: Supplement 1. — eMethods. eReferences. [file jamanetwopen-e2554246-s001.pdf]

Supplemental Online Content

Kim H, Edelstein S, Senders A, et al. Medicare and Medicaid behavioral health service use among dual-eligible special needs plan enrollees. *JAMA Netw Open*. 2026;9(1):e2554246. doi:10.1001/jamanetworkopen.2025.54246

eMethods.

eReferences.

This supplemental material has been provided by the authors to give readers additional information about their work.

© 2026 Kim H et al. *JAMA Network Open*.

eMethods.

Cohort Selection

The following table shows steps taken to create our study cohort.

Selection step	Enrollees <65 (N)	Percent dropped of total (<65)	Enrollees 65+ (N)	Percent dropped of total (65+)
Enrolled in MA, fully dually eligible, residing in US	1,578,879		3,089,407	
Enrolled in a D-SNP plan	1,020,289	35.4%	1,760,812	43.0%
Enrolled in Medicare Advantage complete contracts[a]	975,969	2.8%	1,688,649	2.3%
Has existing TAF record	972,849	0.2%	1,681,234	0.2%
Has same state of residence in Medicare and Medicaid data	956,712	1.0%	1,661,250	0.6%
Has consistent Medicare/Medicaid dual status	941,189	1.0%	1,646,582	0.5%
Not enrolled in hospice	939,552	0.1%	1,632,379	0.5%
Resides in state with good TAF data quality[b]	652,250	18.2%	955,022	21.9%
Has mental health or SUD condition	401,186	15.9%	407,909	17.7%

Note: % dropped of total = portion dropped as a percentage of the original total.
[a]Highly complete Medicare Advantage contracts were defined following Jung et al. (2022)[1] as contracts with at least 2,500 enrollees and fewer than 10 percent missing hospital-stay records. Medicare Advantage encounter data were validated against MedPAR hospitalization records to identify reliable contracts.
[b]States with reliable TAF data quality were identified based on data quality measures from Medicaid and CHIP Data Quality (DQ) Atlas assessments, and additional custom analyses. We used DQ Atlas measures of claims volume for inpatient (IP), outpatient (OT), and long-term care (LT) files, modifying the definition of “high concern” and “unusable” values as those with claims volumes below the national median. We also used created custom assessments to evaluate the completeness and validity of procedure and diagnosis codes from IP, OT, and LT files, including the proportion of missing or invalid CPT/ICD codes.

© 2026 Kim H et al. *JAMA Network Open*.

Methods in Figure

We identified behavioral health service use in Medicare and Medicaid claims using diagnosis, procedure, revenue, and place of service codes (eMethods in Supplement 1). We limited the analysis to claims with a behavioral health diagnosis code in the primary position and excluded Medicaid crossover claims (CROSSOVER_CLM_IND value of 1) and Medicare Advantage encounter records with a procedure modifier code U1-U9 or UA-UD (procedures not covered by Medicare). We used claim line dates to capture when services were rendered and paid by each source. To determine the primary payer, we compared Medicaid and Medicare services occurring on the same claim line dates. A service was considered a match if the claim line shared the same dates, diagnosis code, and procedure or revenue code. When a match occurred, the service was attributed to Medicare as the primary payer; however, residential treatment days were attributed to Medicaid given that Medicare policy was to not cover residential treatment facility stays in 2021. If both Medicare and Medicaid covered different components of a service (eg, dates matched but codes did not), we attributed the service to both payers.

© 2026 Kim H et al. *JAMA Network Open*.

Identification of behavioral health conditions

Mild/moderate and severe mental illness

To identify enrollees with a mental health diagnosis, we first used the Agency for Healthcare Research and Quality's Clinical Classifications Software Refined (CCSR) to identify a broad list of mental health diagnostic codes.[2] We began with all ICD-10 codes included in the CCSR body system "Mental, Behavioral, and Neurodevelopmental Disorders". We then excluded codes related to a physiologic condition, substance use, or childhood condition. Our final set of ICD-10 codes included F20-69 and F90-99 (including subcategory codes and excluding F55, F64.2, F93, F94.8, F94.9, and F98), as well as all the codes in the CCSR Category MBD012 "Suicidal ideation/attempt/intentional self-harm".

We used all relevant Medicare Advantage and Medicaid claims to identify conditions. We defined enrollees with severe mental illness (SMI) as those with at least 1 inpatient OR 2 other non-drug claims of any service type in 2021 with any diagnosis of schizophrenia (F20, F25), bipolar I (F30, F31.0-F31.78), or major depressive disorder (F32.2, F32.3, F33.2, F33.3).[3]

We defined enrollees with mild-to-moderate mental illness as those who did not meet the criteria for SMI and had at least 1 inpatient OR 2 other non-drug claims of any service type in 2021 with a mental health diagnosis code.[3]

Enrollees who did not meet the criteria for SMI or mild-to-moderate illness were categorized as not having a mental health condition.

Substance use disorder (SUD)

To identify enrollees with a SUD diagnosis, we applied algorithms published by the Chronic Conditions Warehouse for alcohol use disorder, drug use disorder, opioid use disorder, and opioid-related hospital or emergency department (ED) visits to Medicaid and Medicare Advantage claims.[4] In brief, we defined enrollees with one inpatient OR two or more non-drug claims of any service type with relevant diagnosis, procedure, and national drug code codes as having SUD.

© 2026 Kim H et al. *JAMA Network Open*.

Identification of behavioral health service use and attribution of payer

We identified behavioral health (BH) service use in Medicare and Medicaid claims using diagnosis, procedure, revenue, and place of service codes (details below). We limited to claims with a behavioral health diagnosis code (as above) in the primary position and excluded Medicaid crossover claims (CROSSOVER_CLM_IND value of 1) and Medicare claims with a procedure modifier code U1-U9 or UA-UD. We used claim line dates to capture when services were rendered and paid for by each source. To determine the primary payer, we compared Medicaid and Medicare services occurring on the same claim line date(s). A service was considered a match if the claim line shared the same date(s), diagnosis code, and procedure and/or revenue code. When a match occurred, the service was attributed to Medicare as the primary payer, with one exception: Residential treatment days were attributed to Medicaid, since Medicare policy was to not cover residential stays in 2021. If both Medicare and Medicaid covered different components of a service (e.g., date(s) matched but codes did not), we attributed the service to both payers.

Using claim line dates in this way improved the precision of payer attribution. However, this approach may slightly undercount inpatient days if header dates spanned more days than the sum of claim line dates.

Outpatient/telehealth visits

We limited to professional claims with CPT codes used during mental health specific or primary care visits, including those for assessment/screening, community support, drug visits, evaluation and management, psychiatry, and psychotherapy. We developed our CPT code list through consultation with subject matter experts and cross-referencing the Milbank Memorial Fund's recommendations on measuring behavioral health spending (Table S1).[5] Telehealth was distinguished from in-person through any of the following: CPT code 99441-99443 or 98966-98968; procedure modifier code 93, 95, FQ, FR, or GT; or place of service code 02 or 10.

Table S1. CPT codes used to identify outpatient/telehealth mental health and substance use disorder services.

Mental health only	J0401, J1631, J1944, J2358, J2426, J2680, J2794
Substance use disorder only	99408, 99409, C9154, G0396, G0397, G0442, G0443, G2011, G2067, G2068, G2069, G2070, G2071, G2072, G2073, G2074, G2075, G2078, G2079, H0001, H0005, H0006, H0007, H0014, H0016, H0020, H0021, H0022, H0026, H0027, H0028, H0029, H0047, H0049, H0050, J0570, J0571, J0572, J0573, J0574, J0575, J0576, J0592, J1230, J2315, Q9991, Q9992, S0109, S9475, T1006, T1007, T1012

© 2026 Kim H et al. *JAMA Network Open*.

Mental health or substance use disorder	90785, 90791, 90792, 90832, 90833, 90834, 90835, 90836, 90837, 90838, 90839, 90840, 90845, 90846, 90847, 90849, 90853, 90863, 90865, 90867, 90868, 90869, 90870, 90875, 90876, 90880, 90899, 96101, 96102, 96103, 96116, 96118, 96119, 96120, 96121, 96127, 96130, 96131, 96132, 96133, 96136, 96137, 96138, 96139, 96146, 96150, 96151, 96152, 96153, 96154, 96155, 96156, 96158, 96159, 96160, 96164, 96165, 96167, 96168, 98966*, 98967*, 98968*, 99201, 99202, 99203, 99204, 99205, 99211, 99212, 99213, 99214, 99215, 99241, 99242, 99243, 99244, 99245, 99341, 99342, 99343, 99344, 99345, 99347, 99348, 99349, 99350, 99354, 99355, 99366, 99385, 99386, 99387, 99395, 99396, 99397, 99401, 99402, 99403, 99404, 99411, 99412, 99415, 99416, 99420, 99429, 99441*, 99442*, 99443*, 99450, 99455, 99456, 99484, 99492, 99493, 99494, 99499, G0074, G0463, G0511, G0512, G2214, H0002, H0004, H0023, H0024, H0025, H0030, H0031, H0032, H0033, H0034, H0036, H0037, H0038, H0039, H0040, H0046, H2000, H2010, H2011, H2014, H2015, H2016, H2017, H2018, H2019, H2020, H2021, H2022, H2023, H2024, H2025, H2026, H2027, H2030, H2031, H2032, H2033, S9482, S9484, S9485, T1015, T1016, T1017, T1023, T1502, T2022, T2023

*CPT was recorded as telehealth. Additionally, any CPT paired with a place of service code 02 or 10 or procedure modifier code 93, 95, FQ, FR, or GT was recorded as telehealth.

Emergency department visits

Any claim with a CPT code for emergency department (99281-99285) or advanced life support (99288), or an emergency department revenue code (450-459 or 981) paired with at least one CPT other than laboratory services.[6]

Inpatient days

We adapted methodology detailed in the Milbank report,[5] which requires either an inpatient revenue code or a combination of inpatient POS code and inpatient CPT codes. We follow this approach but exclude the following codes that would indicate a behavioral health-focused encounter during an inpatient stay but not an inpatient stay for behavioral health purposes: POS codes for nursing facility, hospice, intermediate care, rehabilitation facility, and telehealth; revenue codes for coronary, hospice, nursery, obstetrics, and oncology units; and CPT codes for neonatal, infant, and telehealth care. We count any days covered by the line dates of valid claims.

Residential days

We followed the Milbank report methodology with the same adaptations (where relevant) as were made for inpatient days. Additionally, we excluded codes for lower levels of care, including supervised living, halfway house, group home, and outdoor/wilderness behavioral health. We count any days covered by the line dates of valid claims.

© 2026 Kim H et al. *JAMA Network Open*.

eReferences.

1. Jung J, Carlin C, Feldman R, Tran L. Implementation of resource use measures in Medicare Advantage. *Health Services Research*. 2022;57(4):957-962. doi:10.1111/1475-6773.13970

2. Clinical Classifications Software Refined (CCSR) for ICD-10-CM Diagnoses. Accessed April 1, 2022. https://www.hcup-us.ahrq.gov/toolssoftware/ccsr/dxccsr.jsp

3. McConnell KJ, Edelstein S, Hall J, et al. Access, Utilization, and Quality of Behavioral Health Integration in Medicaid Managed Care. *JAMA Health Forum*. 2023;4(12):e234593. doi:10.1001/jamahealthforum.2023.4593

4. Other Chronic Health, Mental Health, and Potentially Disabling Conditions. Chronic Conditions Data Warehouse. Accessed April 10, 2024. https://www2.ccwdata.org/condition-categories-other

5. Sinha V, Rourke E, Condon MJ, Brandel W. *Recommendations for a Standardized State Methodology to Measure Clinical Behavioral Health Spending*. Milbank Memorial Fund & Freedman HealthCare; 2024.

6. Handel DA, McConnell KJ, Wallace N, Gallia C. How much does emergency department use affect the cost of Medicaid programs? *Ann Emerg Med*. 2008;51(5):614-621, 621.e1. doi:10.1016/j.annemergmed.2007.09.002

© 2026 Kim H et al. *JAMA Network Open*.
