# Supplementary material for: Medicare and Medicaid Behavioral Health Service Use Among Dual-Eligible Special Needs Plan Enrollees
Source: JAMA Netw Open. 2026 Jan 15;9(1):e2554246. doi: 10.1001/jamanetworkopen.2025.54246 (PMC12809357; doi:10.1001/jamanetworkopen.2025.54246)
Supplement: Supplement 2. — Data Sharing Statement [file jamanetwopen-e2554246-s002.pdf]

Data Sharing Statement

Kim. Medicare and Medicaid Behavioral Health Service Use Among Dual-Eligible Special Needs Plan Enrollees. *JAMA Netw Open*. Published online January 15, 2026. doi:10.1001/jamanetworkopen.2025.54246

Data
Data available: No
